# Functional Divergence of the Arg/N‐Degron Pathway Between the Crop *Brassica rapa* and the Model Plant *Arabidopsis thaliana*

**DOI:** 10.1002/pld3.70158

**Published:** 2026-03-19

**Authors:** Brian C. Mooney, Pablo Garcia, Shreenivas Kumar Singh, Emmanuelle Graciet

**Affiliations:** ^1^ Department of Biology Maynooth University Maynooth Ireland

**Keywords:** arginine‐transferase, *Brassica rapa*, hypoxia, N‐degron pathway, PROTEOLYSIS 6, stress

## Abstract

The ubiquitin‐dependent Arg/N‐degron pathway relates the stability of a substrate protein to the nature of its N‐terminal amino acid residue or its biochemical modifications, with some N‐terminal residues being recognized by specific E3 ubiquitin ligases, resulting in the ubiquitylation and degradation of the substrate protein. Work in the model plant *Arabidopsis thaliana* has shown that the Arg/N‐degron pathway is a key regulator of plant responses to hypoxia, which can be either physiological or a stress in the context of waterlogging or submergence. The role of the Arg/N‐degron pathway in hypoxia response is mediated via the oxygen‐dependent degradation of group VII ETHYLENE RESPONSE FACTOR (ERFVII) transcription factors, which act as the master regulators of the hypoxia response program in plants. Analysis of Arabidopsis mutants for different enzymatic components of the Arg/N‐degron pathway has also revealed its roles in the regulation of responses to other abiotic stresses (e.g., salt stress), as well as to pathogens. Although much has been learned from studies in Arabidopsis about the functions of the Arg/N‐degron pathway, very little is known about this pathway in crops, including in Brassica crops such as oilseed rape, cabbage, or turnip. To determine functional similarities and divergence of the Arg/N‐degron pathway between Arabidopsis and Brassica crops, we isolated and characterized the first Arg/N‐degron pathway mutants in *Brassica rapa* (turnip, pak choi), a diploid Brassica crop closely related to oilseed rape. We focused on two enzymatic components, namely, the arginine‐transferases (*ATE*s) and the E3 ubiquitin ligase *PROTEOLYSIS6* (*PRT6*). Our results show both similarities and divergence of function for these Arg/N‐degron pathway components in *B. rapa* compared to Arabidopsis. Specifically, *ATE* mutants in *B. rapa* arrest their development at the seedling stage, which contrasts with the mild phenotypic defects of the equivalent Arabidopsis mutants. Double mutant lines for two of the three *PRT6* genes in *B. rapa* indicated a constitutive activation of hypoxia response genes at the transcriptional level, as shown in the single *prt6* mutant in Arabidopsis. However, contrary to Arabidopsis, the *B. rapa* double mutants were more sensitive to waterlogging and hypoxia and did not show differential response to salt stress or to biotic stress compared to the wild type. The functional divergence identified likely reflects variability in each species in the substrate repertoire and/or in the regulation of pathways or targets downstream of Arg/N‐degron pathway substrates. Such differences could be driven by direct selective pressures at N‐termini (e.g., gain or loss of a destabilizing N‐terminal residue) or by species‐specific proteases that may generate destabilizing neo‐N‐termini after cleavage. These similarities and differences highlight the difficulties in translating research findings from Arabidopsis to crops, even within the same plant family (Brassicaceae), and highlight the need to study pathways in crops.

## 1 Introduction

**FIGURE 1:**
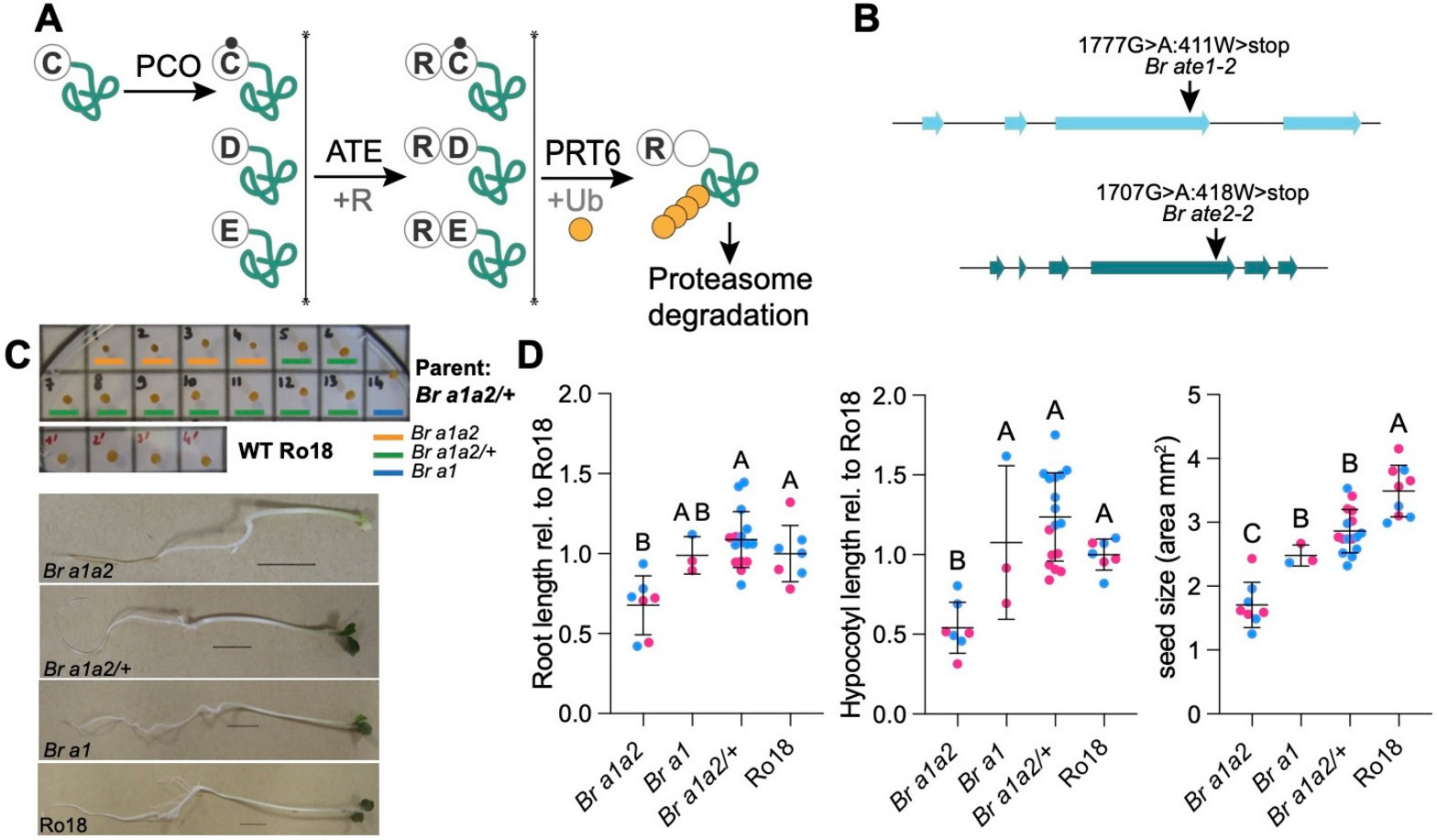
Phenotype of a *Br a1a2* mutant. (A) Overview of the Arg/N‐degron pathway and its enzymatic components. PLANT CYSTEINE OXIDASE (PCO) are oxygen‐dependent enzymes that oxidize the N‐terminal Cys (C) residue of proteins (Weits et al. 2014; White et al. 2017). Their substrates can then be arginylated by ATE enzymes, which also conjugate Arg (R) to substrates with N‐terminal Asp (D) and Glu (E) (Graciet et al. 2009, 2010). Arginylation is followed by recognition and ubiquitylation mediated by the E3 ubiquitin ligase PROTEOLYSIS 6 (PRT6) and degradation by the proteasome (Garzon et al. 2007). PRT6 also recognizes proteins that start with other positively charged residues such as lysine (not depicted) (Garzon et al. 2007). (B) Gene structure of *Br ATE1* and *Br ATE2* and position of the point mutations in *Br ate1‐2* (*Br a1*) and *Br ate2‐2* (*Br a2*). Base pair numbering starts at the ATG of the genomic locus. Amino acid substitutions and their position in the protein are indicated. W, tryptophan. (C) Seed and seedling phenotype in the offspring population of a *Br a1a2*/+ parent compared to the Ro18 wild type. *Br a1a2*: double homozygous mutant; *Br a1a2/+:* homozygous mutant for *Br ATE1* and heterozygous mutant for *Br ATE2*; and *Br a1*: single homozygous mutant for *Br ATE1*. Seedlings were grown under continuous light (20°C) on 0.5× MS agar medium supplemented with 1% (w/v) sucrose. Pictures were taken after 4 days of growth. Scale bar = 1 cm. (D) Quantitation of the phenotypes shown in (C), including seed size area, root length relative to that of the average Ro18, and hypocotyl length relative to the average Ro18. Mean and standard deviations are shown. Statistical analysis: ANOVA and Tukey post hoc test; significant differences: *p* value < 0.05. Pink symbols: data obtained from offspring of parent #5 (*Br a1a2*/+); blue symbols: data from offspring of parent #8 (*Br a1a2*/+). Each symbol represents individual seeds/seedlings from a given biological replicate. Data from two biological replicates.

The ubiquitin/proteasome system plays essential roles in the regulation of plant responses to biotic and abiotic stresses. In plants, the ubiquitin‐dependent N‐degron pathway in particular functions as a key regulator of plant responses to low oxygen conditions (hypoxia) (reviewed in Dissmeyer 2019; Varshavsky 2019), which can be caused by environmental conditions such as flooding (including waterlogging or submergence) (Loreti and Perata 2020; Weits et al. 2021). Work in the model plant *Arabidopsis thaliana* has uncovered the biochemical mechanisms underpinning the role of the N‐degron pathway in mediating response to hypoxia (Gibbs et al. 2011; Licausi et al. 2011; Weits et al. 2014), but much less is known about the roles of this pathway in other plants, especially crops. The N‐degron pathways relate the stability of a protein to the identity of its N‐terminal residue or its posttranslational modifications (reviewed in Dissmeyer 2019; Varshavsky 2019). The so‐called Arg/N‐degron pathway includes a number of enzymatic components that act (sequentially) to modify a substrate's N‐terminal residue and bind so‐called N‐terminal destabilizing residues that can act as a degradation signal (degron) (Figure 1A) (Potuschak et al. 1998; Stary et al. 2003; Garzon et al. 2007; Graciet et al. 2009, 2010). Several *bona fide* Arg/N‐degron pathway substrates have been identified in Arabidopsis (Gibbs et al. 2011; Licausi et al. 2011; Gibbs et al. 2018; Goslin et al. 2019; Weits et al. 2019; Labandera et al. 2021), some of which are degraded in an oxygen‐dependent manner, such as for example LITTLE ZIPPER 2 (ZPR2) and VERNALIZATION2 (VRN2), which regulate developmental processes in the context of physiological hypoxic niches (Gibbs et al. 2018; Weits et al. 2019; Labandera et al. 2021).

The best known substrates of the Arg/N‐degron pathway are a set of five group VII ETHYLENE RESPONSE FACTOR transcription factors (collectively noted ERFVIIs) that act as the master regulators of the transcriptional hypoxia response program (Mustroph et al. 2009; Gibbs et al. 2011; Licausi et al. 2011; Reynoso et al. 2019). Their Arg/N‐degron‐dependent degradation renders this pathway a key regulator of hypoxia responses and a component of the oxygen sensing mechanisms in plants. Arabidopsis *prt6* (*proteolysis 6*) mutants, which are affected for the E3 ubiquitin ligase that ubiquitylates the ERFVIIs, exhibit increased tolerance to waterlogging and to hypoxia due to the constitutive accumulation of the ERFVIIs (Gibbs et al. 2011). Notably, Arabidopsis *prt6* mutants also exhibit increased tolerance to other abiotic stresses such as high salt and drought (Vicente et al. 2017). This phenotype is conserved in barley ( *Hordeum vulgare* ) plants expressing an RNAi construct targeting *Hv PRT6*, suggesting that Arg/N‐degron pathway components could be targets of interest to improve crop tolerance to waterlogging and potentially to other abiotic stresses (Mendiondo et al. 2016; Vicente et al. 2017), including osmotic stress (Papdi et al. 2015). In line with this idea, the overexpression of ERFVII homologs in crop species such as maize (Yu et al. 2019) and wheat (Wei et al. 2019) results in improved tolerance to waterlogging. Overexpression in Arabidopsis of the *RAP2.2* ERFVII transcription factor also led to improved resistance to the fungal necrotrophic pathogen *Botrytis cinerea*, suggesting that the function of these ERFVIIs may extend to the regulation of plant defenses against pathogens (Zhao et al. 2012), perhaps due to the formation of local hypoxic niches at the site of *B. cinerea* infection (Valeri et al. 2021). Similarly, overexpression in Arabidopsis of the barley *RAP2.2* homolog (noted *Hv RAF*) also resulted in increased resistance to the bacterial pathogen *Ralstonia solanacearum* (Jung et al. 2007). Notably, Arabidopsis mutants for Arg/N‐degron pathway components also showed differential defense responses to a range of pathogens (de Marchi et al. 2016; Gravot et al. 2016; Vicente et al. 2019). Despite this knowledge and potential for applications of agronomic interest, thus far, very little is known about the roles of the Arg/N‐degron pathway in crops and the properties of crop plants deficient for some of its components.

As a member of the *Brassicaceae* family, Arabidopsis is related to a range of economically important crop species, including *Brassica napus* (oilseed rape), *Brassica oleracea* (cabbage and broccoli), and *Brassica rapa* (e.g., pak choi, turnip, and some oil varieties). *B. rapa* may be considered a more attractive representative model species for Brassica crops because (i) its diploid genome is less complex than that of the allotetraploid *B. napus* ; (ii) the *B. rapa* Chiifu‐401‐42 genome was sequenced over 10 years ago (Wang et al. 2011); (iii) a TILLING (Targeting Induced Local Lesions In Genomes) collection of EMS mutants is available (Stephenson et al. 2010); and (iv) transient expression methods have been optimized (Mooney and Graciet 2020). Here, we sought to establish *B. rapa* as a model system to investigate the functions of the Arg/N‐degron pathway in Brassicaceae crops. Our earlier work revealed that the enzymatic components of the Arg/N‐degron pathway identified in Arabidopsis are conserved in *B. rapa* , alongside additional homologs (Mooney and Graciet 2020). For example, PRT6 is encoded by a single gene in Arabidopsis but by three genes in *B. rapa* . However, similarly to Arabidopsis, *B. rapa* codes for two Arg‐transferases. For the first time, we isolated and characterized *B. rapa* mutants for Arg/N‐degron pathway components (specifically, Arg‐transferases and PRT6) from the Ro18 TILLING population (Stephenson et al. 2010) and showed that they have both similar and divergent phenotypes to their Arabidopsis counterparts. These differences could be relevant to the discovery of new functions of the Arg/N‐degron pathway in plants and to efforts to translate findings from Arabidopsis to Brassica crops.

## 2 Materials and Methods

### 2.1 Plant Growth Media and Conditions

*B. rapa* plants were grown on a sterilized soil mixture containing a 5:3:2 ratio of compost, vermiculite, and perlite. For experiments involving seedlings, *B. rapa* was grown in Petri‐dishes or sterile plastic cups containing 0.5× Murashige and Skoog (MS) medium (pH 5.7) with 6 g/L agar and 0.5% (w/v) sucrose unless stated otherwise in the figure legends. Trays or plates were incubated in the dark at 4°C for 3 days prior to transfer to growth rooms. Plants were grown either in continuous light or in short‐day conditions (8 h light/16 h dark), as specified below.

### 2.2 *B. rapa* Lines Isolated

*B. rapa* subsp. trilocularis (Yellow Sarson) genotype Ro18 was used. Arg/N‐degron mutant lines were isolated from a TILLING population derived from Ro18 EMS mutagenesis (Stephenson et al. 2010) (Table S1). In this collection, about one mutation per 60 kb is expected (Stephenson et al. 2010). Seeds were obtained from RevGen UK (John Innes Centre, Norwich). The *Br ate1‐2, Br prt6.2‐12*, and *Br prt6.3‐1* lines were backcrossed twice to the wild‐type Ro18 parent and *Br ate2‐2* was backcrossed once prior to crossing to *Br ate1‐2*. After crossing, F1 heterozygous plants for both genes were allowed to self‐fertilize in order to obtain segregating populations that could be used to screen for mutant combinations.

### 2.3 Genotyping of Arg/N‐Degron Mutant *B. rapa* Lines

Genomic DNA was extracted as in (Edwards et al. 1991). The *Br ate1‐2, Br ate2‐2*, and *Br prt6.3‐1* single nucleotide polymorphisms (SNPs) were genotyped using Sanger sequencing following amplification of the relevant genomic region by PCR using oligonucleotides BM28/BM29, BM30/BM31, and BM36/BM37, respectively (see Table S2 for oligonucleotide sequences). A dCAPS assay was used to identify the *Br prt6.2‐12* SNP: A nested PCR was carried out using the BM93/BM94 oligonucleotides for the external PCR and BM97/BM98 for the internal PCR, followed by digestion with *Bcl*I for 6 h at 55°C (wild type: 219 bp; *Br prt6.2‐12*: 199 and 20 bp). Presence of the mutation was confirmed by Sanger sequencing after PCR with BM34/BM35.

### 2.4 Reverse Transcription Quantitative PCR (RT‐qPCR)

Total RNA was extracted using the Spectrum Plant Total RNA Kit (Merck). Reverse transcription reactions were set up using 100–1000 ng of isolated total RNA using RevertAid Reverse Transcriptase (Thermo Fisher), RiboLock RNase inhibitor (Thermo Fisher), and oligo (dT)18. qPCR reaction mixtures were set up in 96‐well plates (Roche) with 1 μL of cDNA mixed with 1 μL of a primer pair mixture (1 μM final concentration each) and 5 μL 2X SYBR green master mix (Roche), with nuclease‐free water added to a final volume of 10 μL per well. qPCR reactions were carried out in a LightCycler 480 instrument (Roche). The second derivative maximum method was used to determine crossing point (Cp) values. Gene expression was calculated relative to a reference gene with the comparative Ct method (Cp_reference gene_ − Cp_gene of interest_ = deltaCp). Assuming a PCR efficiency value of 2, relative expression was calculated as 2^deltaCp^. *Br GAPDH* (*GLYCERALDEHYDE 3‐PHOSPHATE DEHYDROGENASE*; Bra016729) was used as a reference gene for RT‐qPCRs in *B. rapa* (Procko et al. 2014). Oligonucleotides used for qPCR are listed in Table S2.

### 2.5 Agroinfiltration and Transient Expression in *B. rapa*

This procedure was carried out as described in (Mooney and Graciet 2020). *A. tumefaciens* C58 pGV2260 (McBride and Summerfelt 1990) transformed with the indicated Arg/N‐degron pathway reporters (Graciet et al. 2010) or a pML‐BART empty vector were grown for 3–4 days at 28°C on LB agar supplemented with 50 mg/L rifampicin, 100 mg/L ampicillin, and 100 mg/L spectinomycin. After 3–4 days growth, bacteria were suspended from plates in 2‐mL infiltration medium (10‐mM MES pH 5.5, 10‐mM MgCl_2_, and 150‐μM acetosyringone) and diluted to OD_600_ of 0.75. Four‐ to five‐week‐old *B. rapa* plants were covered with plastic lids overnight prior to infiltration. An ~2 cm diameter area was marked on the abaxial side of the first and second true leaves. Using a blunt 1‐mL syringe, the bacterial suspension was infiltrated into the marked areas. Excess liquid was removed with tissue paper, and plants were returned to the growth room. Tissue was harvested 3 days postagroinfiltration for protein or RNA extraction.

### 2.6 LUC and GUS Activity Assays With Arg/N‐Degron Pathway Reporter Constructs

All Arg/N‐degron pathway reporter constructs used in this study have been previously published in Worley et al. (1998) and Graciet et al. (2010). The same protocol as in Mooney and Graciet (2020) was used. Briefly, proteins were extracted from frozen ground tissue using 1X Luciferase Cell Culture Lysis Reagent (Promega), supplemented with 1‐mM phenylmethylsulfonyl fluoride (PMSF) and 1:100 plant Protease Inhibitor Cocktail (Merck). Samples were centrifuged at 12,000×*g* for 10 min at 4°C to pellet cellular debris. Protein concentration was determined using the Bradford protein assay.

LUC activity was measured as in Luehrsen et al. (1992), Graciet et al. (2010), and Mooney and Graciet (2020). Briefly, CCLR protein extract (1–2 μL) was added to 100 μL LAR buffer (20‐mM tricine, pH 7.8, 1.07‐mM (MgCO_3_)_4_, Mg (OH)_2_.5H_2_O, 2.67‐mM MgSO_4_, 0.1‐mM ethylenediaminetetraacetic acid (EDTA), 33.3‐mM dithiothreitol (DTT), 270‐μM coenzyme A, 470‐μM luciferin, and 530‐μM ATP) in a 96‐well plate (Sterilin). Luminescence was measured using a POLARstar Omega microplate reader (BMG LABTECH) for 10 s. Luminescence values were then normalized to the relative expression of the *LUC* gene as determined by RT‐qPCR from the same tissue (see Table S2 for oligonucleotide sequences).

### 2.7 *B. rapa* flg22 Treatment for RNA‐Seq

*B. rapa* seedlings were grown in continuous light conditions at 20°C in cups containing 0.5× MS agar supplemented with 0.5% (w/v) sucrose. After 3 days, four seedlings per genotype per treatment were transferred to a well of a six‐well plate containing 6 mL of 0.5× MS supplemented with 0.5% sucrose (liquid medium) and returned to the growth room for incubation overnight with mild shaking. On Day 4, seedlings were treated with 1‐μM flg22 or an equivalent volume of water (mock treatment) for 1 h. Seedlings were collected and frozen immediately in liquid nitrogen prior to RNA extraction with Spectrum Plant Total RNA Kit (Merck). Samples from three biological replicates were used.

### 2.8 RNA‐Seq, Data Processing, and Functional Analysis

RNA integrity was assessed using an Agilent 2100 Bioanalyzer (Agilent). All RNA samples had RNA integrity (RIN) values > 7.0. Library preparation, and paired‐end 100‐bp next‐generation sequencing was performed by BGI (Hong Kong) using the DNB‐seq platform. Data processing was carried out by BGI using the filtering software SOAPnuke (including removal of reads containing the adaptor; removal of reads whose N content is greater than 5%; and removal of low‐quality reads). The Hierarchical Indexing for Spliced Alignment of Transcripts (HISAT2) software was then used for mapping clean reads to the *B. rapa* reference genome available at the time of the study (GCF_000309985.2). Differential gene expression was determined using DESeq2. Cut‐offs of adjusted *p* value < 0.001 and |log_2_ of fold‐change| > 1 were applied to determine differentially expressed genes (DEGs). Significant GO category enrichment analyses of DEGs were carried out using ShinyGO 0.85 (Ge et al. 2020) using the STRING.51351 database as reference (STRING v11.5). The following settings were used: FDR cut‐off = 0.05, minimum pathway size = 2, maximum pathway size of 5000, and Top 25 GO categories, with removal of redundancy and abbreviation of pathways. As recommended in Wijesooriya et al. (2022), background for GO analyses with DEGs in the WT#67^flg/m^ dataset, we used the complete set of genes identified in WT#67 when no cut‐offs were applied (corresponding to 36,534 genes; see Dataset S1). For GO analyses with DEGs in *Br prt6.2/3#68* ^flg/m^, the background used corresponded to all the genes identified in *Br prt6.2/3#68* prior to applying cut‐offs (36,351 genes; see Dataset S2). Overlap between datasets was determined using InteractiVenn (Heberle et al. 2015), and statistical significance of the overlap between datasets was calculated using 2 × 2 contingency tables and Chi‐square tests. Raw and processed data are submitted to NCBI Gene Expression Omnibus under accession number GSE311966.

### 2.9 Waterlogging Stress Experiments

Seeds were sown on sterilized soil, and pots were placed in a tray covered with a transparent lid. After 3 days at 4°C, the trays were transferred to the greenhouse (16 h light/8 h dark). After 1 week of growth, the lids were removed, and the plants were grown for 3 weeks, at which point waterlogging treatment was applied by keeping water approximately 1 cm above the soil level. After 2 weeks of waterlogging, SPAD measurements were taken on two different locations (each side of the midvein) of Leaf 3 using a Multispeq device (PhotosynQ).

### 2.10 Hypoxia Treatment and Chlorophyll Content Determination

Seeds were surface‐sterilized using the vapor‐phase sterilization method (Lindsey et al. 2017) and sown in plastic glasses with 0.5× MS agar medium. Seeds were germinated, and seedlings were grown in continuous light. Seven‐day‐old seedlings were treated with hypoxia by placing the cups into anaerojars with an anaerogen sachet (Oxoid) for 16 h in the dark. Seedlings were then returned to continuous light conditions. After 24 h of recovery, fresh weight of the seedlings was determined and pictures were taken for scoring. Chlorophyll was then extracted following the protocol described in (Sumanta et al. 2014). Scores were defined as 1 = dead seedling (completely white); 2 = seedling more than 40% yellow or white; 3 = seedling approximately 50% white or yellow; 4 = seedling less than 40% yellow or white; and 5 = seedling is completely green. Five biological replicates were performed with four seedlings per genotype, condition, and replicate.

### 2.11 Salt Stress Response Assay

Seeds were surface sterilized using the bleach vapor method (Lindsey et al. 2017).

Sterilized seeds were sown on Petri dishes with 0.8% water agar and the plates were kept for 24 h at 20°C in the dark. Seeds were then transferred to fresh 0.8% water agar plates supplemented with NaCl at concentrations of 100, 150, and 200 mM. As a control, seeds were transferred to a fresh 0.8% water agar plate without NaCl. Plates were then kept vertical in continuous light conditions. Seedlings were photographed at 24, 72, and 96 h posttransfer, and root lengths were measured using ImageJ (Schneider et al. 2012). For each genotype and treatment, five seedlings were used, and three independent biological replicates were performed.

### 2.12 Inoculation With *Sclerotinia sclerotiorum*

A *S. sclerotiorum* sclerotium isolated from an oilseed rape field in Ireland was used to propagate *S. sclerotiorum*. Subsequently, sclerotia were cultured on Potato Dextrose Agar (PDA; pH 5.2–5.5) and incubated at 21°C for 3 days. Actively growing mycelial plugs were then excised using a sterilized cork borer from the leading edge of the colony and transferred to fresh PDA plates for subculturing. The plate was kept for an additional 48 h at 21°C to obtain growing mycelia for inoculation.

For infection assays, fully expanded third leaves of *B. rapa* plants were used. Mycelial agar plugs were excised from the colony margin and placed onto the adaxial surface of the detached leaves. Inoculated leaves were then placed on 90 mm^2^ Petri plates containing 0.8% water agar to maintain humidity. Plates containing inoculated leaves were incubated in short‐day conditions at 21°C to facilitate infection. Necrotic lesions on the leaves were photographed 24 h postinoculation (hpi). Lesion size was measured using the ImageJ software from images acquired from five biological replicates, each containing eight plants per treatment and per biological replicate.

### 2.13 Measurement of Apoplastic Reactive Oxygen Species (ROS)

*B. rapa* was grown at 20°C for 4 weeks in short‐day conditions. Disks (1‐cm diameter) were taken from leaves of 4‐week‐old plants with a cork borer. Leaf disks were then carefully divided into four quarters with a razor blade. Each quarter disk was placed into a separate well of a white Sterilin 96‐well plate (ThermoScientific) containing 200‐μL dH2O with the abaxial leaf surface facing upwards. The plate was then returned to the growth room for a recovery period of at least 3 h. Stock solutions of luminol (Merck) at 100× concentration (17.7 mg/mL in 200 mM KOH) and horseradish peroxidase (HRP) (Fisher Scientific) (10 mg/mL in dH2O) were prepared fresh. Sixty microliters of a luminescence solution containing 2.8‐μL 100× luminol, 2.8‐μL 100× HRP, and 54.4‐μL dH2O was added to each well. The plate was then transferred to a POLARstar Omega microplate reader (BMG LABTECH), and luminescence was detected for 15 min to establish a baseline measurement. During this time, a 1.4‐μM stock solution of flg22 was prepared in dH2O. Twenty microliters of a 1.4‐μM stock flg22 solution was added to each well, bringing the total volume to 280 μL, resulting in final concentrations of 100‐nM flg22, 1× luminol, and 1× HRP. Luminescence was detected every 120 s for a 60‐min period after addition of flg22.

### 2.14 Growth Inhibition Assays in the Presence of flg22

*B. rapa* seedlings were grown in continuous light conditions at 20°C in cups containing 0.5× MS agar supplemented with 0.5% (w/v) sucrose. After 3 days, three seedlings per genotype per treatment were transferred to a well of a six‐well plate containing 6 mL of 0.5× MS with 0.5% sucrose (liquid medium) supplemented with 100‐nM flg22 or an equivalent volume of deionized water (mock). Seedlings were then grown in this liquid culture with mild shaking in continuous light at 20°C for 7 days, at which point seedlings were weighed.

### 2.15 Statistical Analyses

Statistics tests are presented in the figure legends for each of the relevant panels. All statistical tests were performed using GraphPad Prism.

## 3 Results

### 3.1 Mutation of Arg‐Transferases in *B. rapa* Causes Developmental Arrest

One of the best studied Arg/N‐degron pathway mutants in Arabidopsis is the *ate1‐2 ate2‐1* double mutant (noted *a1a2*), which is affected for the two functionally redundant Arg‐transferases *AtATE1* and *AtATE2* (Graciet et al. 2009). Similarly to Arabidopsis, *B. rapa* codes for two Arg‐transferase homologs, *Br ATE1* and *Br ATE2*. Mutant alleles with a premature stop codon were identified for both genes in a TILLING collection generated in the Ro18 background (Stephenson et al. 2010). Specifically, the *Br ate1‐2* mutation substitutes a tryptophan residue at amino acid 411 with a stop codon (TGG → TAG), whereas *Br ate2‐2* presents a stop codon (TGG → TGA) instead of a tryptophan at amino acid 418 (Figure 1B). After backcrossing each of these mutant lines with the parental Ro18 genotype to reduce the impact of background mutations, *Br ate1‐2*/+ and *Br ate2‐2*/+ heterozygous mutant plants were crossed to each other. The F2 population was screened for a *Br ate1‐2 ate2‐2* double homozygous mutant (noted *Br a1a2*), but no double mutant could be identified. In the F2 population, single homozygous mutant *Br ate1‐2* and *Br ate2‐2* could be isolated (Figure S1), as well as *Br ate1‐2/+ ate2‐2* (heterozygous for *Br ATE1* and homozygous mutant for *Br ATE2*) and *Br ate1‐2 ate2‐2/+* (homozygous mutant for *Br ATE1* and heterozygous mutant for *Br ATE2*) plants. When F3 seeds from the *Br ate1‐2/+ ate2‐2* parent were sown, 20 plants grew, 10 of which were *Br ate2‐2* single mutants and 10 were *Br ate1‐2/+ ate2‐2*. Next, seeds from two individual *Br ate1‐2 ate2‐2/+* plants were collected, and the segregating F3 populations obtained from these two *Br ate1‐2 ate2‐2*/+ parents were grown on 0.5× MS plates supplemented with 0.5% sucrose. Seedlings displaying underdeveloped, white cotyledons were consistently identified as double homozygous *Br a1a2* mutants by genotyping and represented about ¼ of the populations (Figure 1C). Other seedlings in the segregating population were either *Br ate1‐2* or *Br ate1‐2 ate2‐2/+*. The *Br a1a2* seedlings had shorter hypocotyls and roots compared to the Ro18 parent, *Br ate1‐2* or *Br ate1‐2 ate2‐2/+* seedlings (Figure 1D). Matching genotype and seed morphology further revealed that seeds of the *Br a1a2* double mutants were smaller than those of genotypes, which retained at least one functional copy of *Br ATE2* (Figure 1C,D), even though *Br ate1‐2 ate2‐2/+* seeds were also smaller than those of the Ro18 parental genotype. When left on 0.5× MS medium supplemented with 0.5% sucrose, *Br a1a2* seedlings did not develop beyond the seedling stage. Altogether, these data suggest that, in *B. rapa* , Arg‐transferases likely play functionally redundant essential roles in early development. Further characterization of *Br a1a2* plants, both phenotypically and biochemically, was not possible.

### 3.2 Isolation and Characterization of a *B. rapa* *prt6.2/3* Double Mutant

**FIGURE 2:**
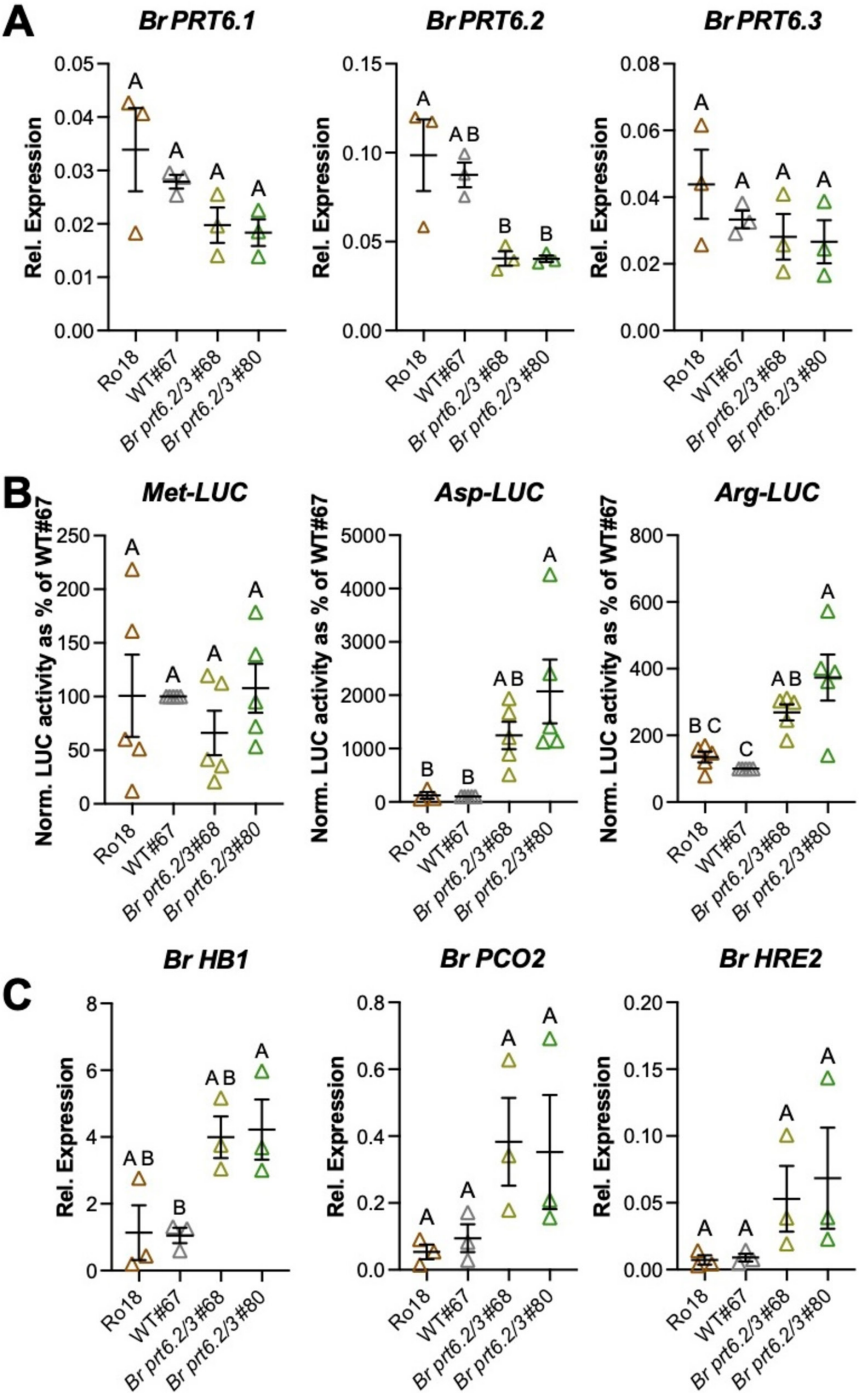
Accumulation of Br PRT6 substrates in *Br prt6.2/3* double mutant lines. (A) Expression of *Br PRT6.1*, *Br PRT6.2*, and *Br PRT6.3* in 3‐day‐old seedlings of the wild type and *Br prt6.2/3* mutant. Expression was determined using RT‐qPCR. The individual relative expression values to the reference gene (*Br GAPDH*) for three biological replicates are shown, together with the mean and standard error of the mean (SEM). Statistical significance (*p* value < 0.05) is presented using the CLD format following one‐way ANOVA and Tukey's test. (B) Arg/N‐degron pathway reporter constructs stability in wild type and *Br prt6.2/3* double mutant lines in transient expression assays. LUC activities normalized for LUC transcript levels were determined and are presented as a percentage of the activity in the WT#67 line. Data represent mean and SEM of five biological replicates. Statistical significance (*p* value < 0.05) is presented using the CLD format following one‐way ANOVA and Tukey's test. (C) Expression of hypoxia response marker genes in 3‐day‐old wild type and *Br prt6.2/3* seedlings grown on 0.5× MS supplemented with 0.5% sucrose plates. Relative expression levels to the *Br GAPDH* reference gene were determined using RT‐qPCR. Mean and SEM of three biological replicates are shown. Statistical significance (*p* value < 0.05) is presented using the CLD format following one‐way ANOVA and Tukey's test.

A previous BLASTp analysis identified three potential homologs of Arabidopsis *PRT6* in *B. rapa* (noted, *Br PRT6.1*, *Br PRT6.2*, and *Br PRT6.3*) (Mooney and Graciet 2020), with *Br PRT6.2* being more highly expressed in wild‐type Ro18 seedlings than *Br PRT6.1* and *Br PRT6.3* (Figure 2A). TILLING mutant alleles with premature stop codons for each of these *Br PRT6* homologs were identified (Table S1). To avoid an early developmental arrest phenotype similar to that observed in *Br a1a2* mutants, we instead isolated a *Br prt6.2 prt6.3* double mutant (noted *Br prt6.2/3*) that retained the function of the lesser‐expressed *Br PRT6.1*. The *Br prt6.2‐12* and *Br prt6.3‐1* mutant alleles were selected because of the presence of premature stop codons at amino acids 1579 (out of 1986) and 1329 (out of 1968), respectively. These single mutants were back‐crossed into the Ro18 parental line, and heterozygous individuals were then crossed to each other to isolate a *Br prt6.2/3* double mutant. Two double homozygous mutant lines (noted #68 and #80) were identified in the F2 population. Within the same segregating F2 population, a line wild‐type for both *Br PRT6.2* and *Br PRT6.3* (noted WT#67) was also isolated as an additional “wild‐type” control for the presence and potential effects of segregating background mutations that may have been retained despite back‐crossing with Ro18. Unlike *Br a1a2* double mutant plants, the *Br prt6.2/3* double mutant lines exhibited a wild‐type‐like ontogeny.

Analysis of the expression of *Br PRT6.1*, *Br PRT6.2*, and *Br PRT6.3* in the different genetic backgrounds indicated that *Br PRT6.2* mRNA levels were significantly decreased in the *Br prt6.2/3* double mutants, whereas the mRNA levels of *Br PRT6.1* and *Br PRT6.3* remained similar in the double mutant or in the two wild‐type backgrounds used as a control (Figure 2A). Critically, transient expression of Ub‐X‐LUC Arg/N‐degron pathway reporters in leaves of double mutant (*Br prt6.2/3* #68 and #80) and wild‐type (Ro18 and WT#67) plants showed significantly increased accumulation of Arg‐LUC and Asp‐LUC in each of the *Br prt6.2/3* double mutant lines compared to the two wild type lines, whereas Met‐LUC stability was the same irrespective of the genetic background (Figure 2B). To further confirm the disruption of Arg/N‐degron pathway function in *Br prt6.2/3*, we tested whether hypoxia response genes were constitutively upregulated in *Br prt6.2/3* seedlings, as would be expected from constitutive accumulation of *B. rapa* ERFVII transcription factor homologs, and found that the expression of *B. rapa* hypoxia response marker genes such as *Br HB1* (Bra001958), *Br PCO2* (Bra025636), and *Br HRE2* (Bra021401) was higher in average (although not always statistically significant) in both *Br prt6.2/3* double mutant lines (#68 and #80) compared to the two wild types (Ro18 and #67) (Figure 2C). Altogether, PRT6 activity in *Br prt6.2/3* double mutant plants is sufficiently disrupted to allow accumulation of artificial and natural Arg/N‐degron pathway substrates.

### 3.3 Abiotic Stress Responses of *Br prt6.2/3* Double Mutant

**FIGURE 3:**
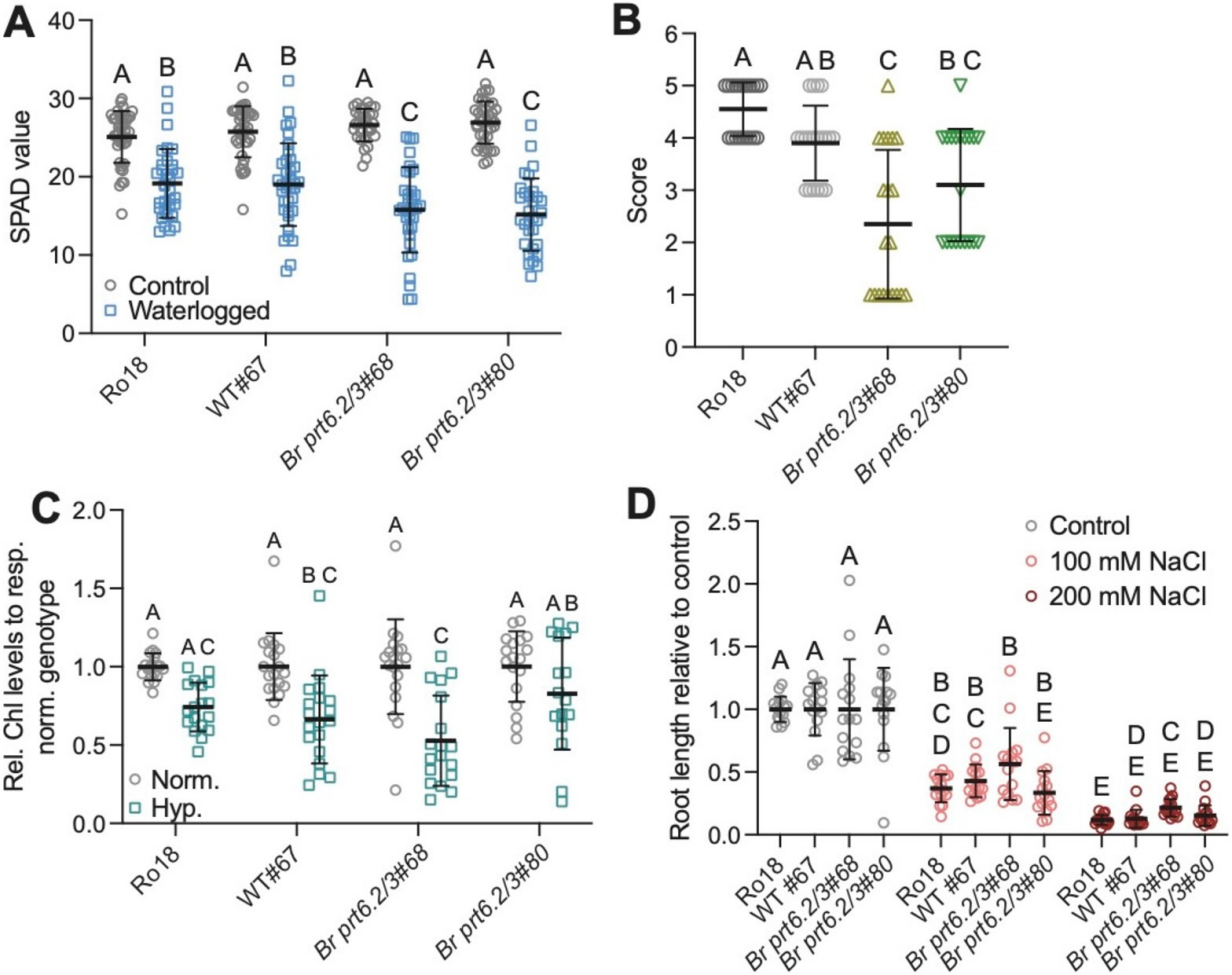
Response of the *Br prt6.2/3* mutant to abiotic stresses. (A) SPAD values obtained after a 14‐day waterlogging treatment or of control plants kept under normal watering conditions. Mean with standard deviations (SD) of four biological replicates (eight plants per treatment per biological replicate) are shown. Statistical significance was assessed by two‐way ANOVA with Sidak's multiple comparison test. (B) Score of seedling health following hypoxia treatment for 16 h in the dark, followed by a 24‐h recovery period. Mean scores with SD of five biological replicates are shown (four seedlings per biological replicate). Statistical significance was assessed using one‐way ANOVA, followed by Tukey's test. (C) Total chlorophyll (chl; corresponding to chla + chlb) levels relative to those in the same genotype left under normoxic conditions. Mean and SD are shown from five biological replicates with four seedlings per biological replicate (except Replicate 1, which had only two seedlings). Two‐way ANOVA with Sidak's multiple comparison test was used to assess statistical significance of differences. (D) Root length (in cm) of seedlings grown for 72 h with 100‐ or 200‐mM NaCl, or mock‐treated. For each genotype and treatment, five seedlings were used, and three biological replicates were performed. Two‐way ANOVA with Tukey's multiple comparison test was used to assess statistical significance of differences.

One of the most notable phenotypes of *prt6* mutant plants, both in Arabidopsis and in barley, is an increased tolerance to waterlogging and to hypoxia as a result of the constitutive accumulation of ERFVII transcription factors (Gibbs et al. 2011; Mendiondo et al. 2016). The tolerance to waterlogging of *Br prt6.2/3* was therefore examined. Following 15 days of waterlogging of 3‐week‐old *B. rapa* plants, SPAD measurements were taken as an assessment of relative chlorophyll content. Based on these SPAD values, the waterlogging treatment negatively affected all genotypes, with a stronger negative effect on the two *Br prt6.2/3* mutant lines (Figure 3A). To complement these results, the tolerance of 7‐day‐old seedlings to hypoxic treatment in the dark for 16 h was assessed following a 24‐h recovery period (Figure 3B). The results suggest that the *Br prt6.2/3* double mutant lines are more sensitive to hypoxia than either of the two wild‐type controls. To obtain a more accurate assessment of the effect of hypoxia on the seedlings, total chlorophyll levels were determined (Figure 3C). Apart from *Br prt6.2/3* #80 for which no difference between normoxia and hypoxia treatment was apparent, the three other genotypes were confirmed to be negatively affected by hypoxia treatment. However, it was not possible to identify relative hypoxia tolerance differences.

Arabidopsis *prt6* mutants have also been shown to be more tolerant to salt stress (Vicente et al. 2017). Similar tests with *Br prt6.2/3* mutant lines did not detect any differences between the wild‐type genotypes and the double mutant lines (Figure 3D). In sum, in contrast to Arabidopsis *prt6* mutant seedlings and plants, *Br prt6.2/3* exhibited increased sensitivity to waterlogging and hypoxia, whereas salt stress did not reveal any differences between the genotypes.

### 3.4 Immune and Biotic Stress Responses of *Br prt6.2/3* Double Mutants

**FIGURE 4:**
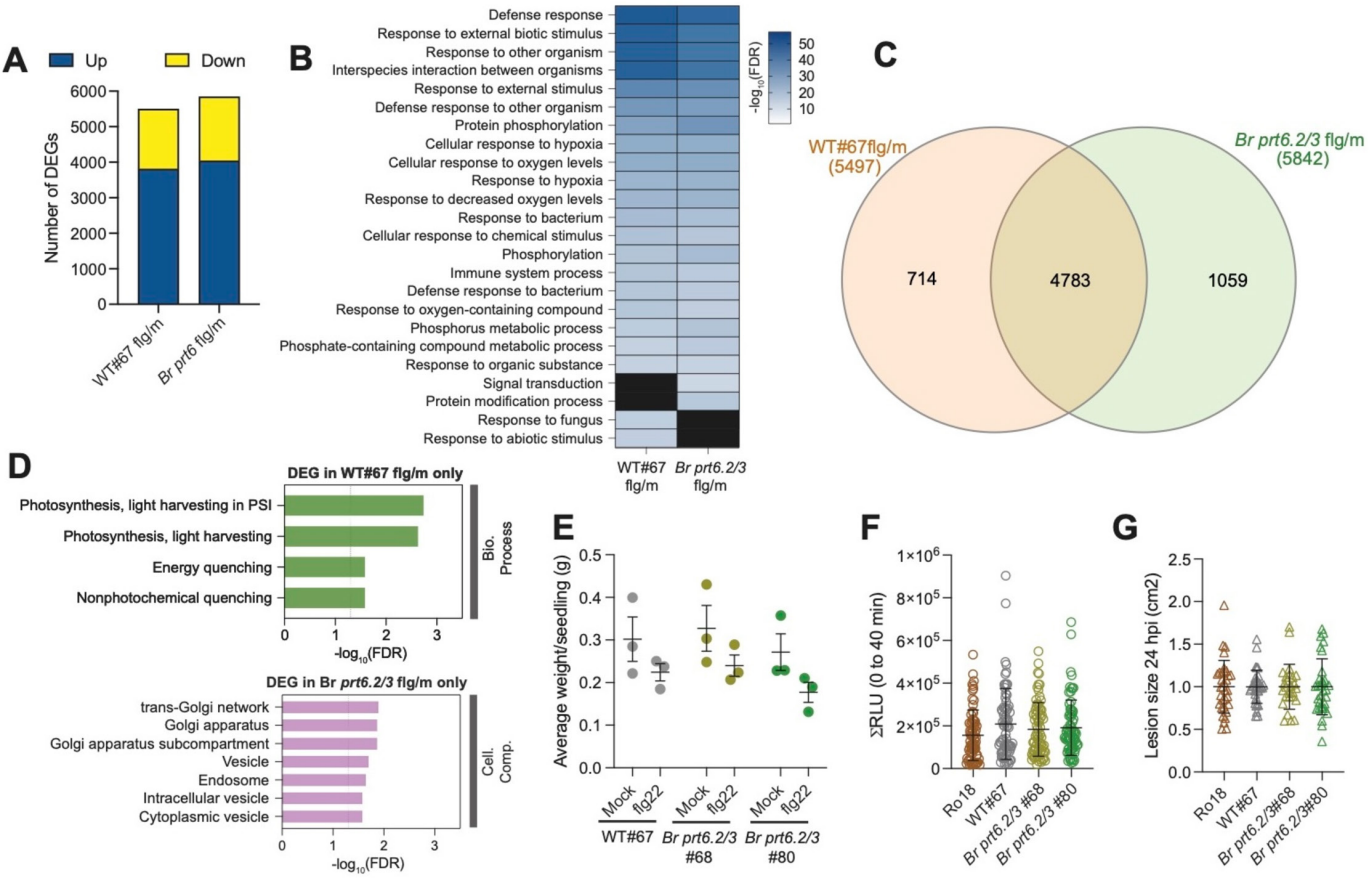
Response of *Br prt6.2/3* mutants to flg22 and *S. sclerotiorum*. (A) Number of DEGs in WT#67 and in *Br prt6.2/3*#68 (*Br prt6*) when comparing flg22‐treated to mock‐treated seedlings (flg/mock) with adj. *p* < 0.001 and |log2FC| > 1.0. (B) Top 25 GO Biological Process terms obtained using ShinyGO v0.85 (FDR < 0.05). The GO term “biological proc. involved in interspecies interaction between organisms” was abbreviated “interspecies interaction between organisms.” (C) Overlap and differences between the WT#67^flg/m^ and in *Br prt6.2/3*#68^flg/m^ DEGs. (D) GO analysis to determine enrichment for “biological process” and also for “cellular compartment” among the 714 DEGs specific to WT#67^flg/m^ (green) and the 1059 DEGs in *Br prt6.2/3*#68^flg/m^ (pink) only (FDR < 0.05). PSI, Photosystem I. (E) Growth inhibition of 3‐day old seedlings cocultivated with 100‐nM flg22 for 7 days. Mean and SEM from three biological replicates are shown. No statistically significant differences after two‐way ANOVA. (F) ROS production assay. Datapoints represent means of 72 readings (leaf‐disk quarters) taken from 18 leaf disks over three independent replicates. Error bars indicate SEM. RLU, relative light units. No statistically significant differences after one‐way ANOVA. (G) Lesion area measured 24 hpi with *S. sclerotiorum*. Mean and SD of five biological replicates are shown with three to eight plants per replicate. No statistically significant differences after one‐way ANOVA.

Arabidopsis Arg/N‐degron pathway mutants have been shown to be affected for their response to a range of pathogens, albeit with different resistance/susceptibility profiles (de Marchi et al. 2016; Gravot et al. 2016; Vicente et al. 2019). In addition, recent findings have shown connections between the transcriptional response programs to hypoxia and to the model pathogen‐associated molecular pattern (PAMP) flg22, which originates from the bacterial flagellin protein and can be used to elicit the first branch of the plant innate immune system, known as pattern‐triggered immunity (PTI) (Mooney et al. 2024). To investigate defense‐related similarities and differences between Arabidopsis and *B. rapa* Arg/N‐degron pathway mutants, innate immune responses of *Br prt6.2/3* double mutants were first compared to those of the two wild‐type genotypes using flg22. Gene expression analysis using RT‐qPCR indicated that treating 3‐day old seedlings with 100‐μM flg22 for 1 h was sufficient to trigger differential expression of two PTI marker genes (*Br MPK3* [Bra038281] and *Br RBOHD* [Bra020724]) in *B. rapa* (Figure S2A). RNA‐seq analysis using the same experimental conditions was performed to compare the transcriptomes of the WT#67 and *Br prt6.2/3* lines. Cut‐off values of adjusted *p* value < 0.001 and |log_2_ of fold‐change| > 1 were applied to determine the sets of differentially expressed genes (DEGs) in flg22‐treated seedlings compared to mock‐treated ones (flg/m) for each genotype. Similar numbers of upregulated (~3900) and downregulated (~1700) genes were identified in WT#67^flg/m^ and in *Br prt6.2/3#68* ^flg/m^ (Figure 4A and Datasets S1 and S2, respectively). Analysis to identify gene ontology (GO) terms that were enriched in each of these two datasets retrieved expected terms, such as for example “defense response” or “response to other organism” (Figure 4B and Datasets S1 and S2). Most of the top 25 GO categories identified were common to both WT#67^flg/m^ and *Br prt6.2/3#68* ^flg/m^ datasets. To further analyze differences in the transcriptional response programs of WT#67 and *Br prt6.2/3#68* to flg22, we determined the overlap between the two datasets and identified a statistically significant overlap (*p* value < 10^−4^; Chi^2^ test; 4783 common DEGs) (Figure 4C). In addition, DEGs common to both datasets showed the same directionality of gene expression change and a similar amplitude of upregulation or downregulation (Figure S2B).

Despite this large overlap, 714 and 1059 genes were differentially regulated in only one of the 2 genotypes, that is, in the WT or in the *Br prt6.2/3* mutant, respectively. This suggests some differences in the flg22 response of WT and *Br prt6.2/3* seedlings. GO Biological Process analysis of the 714 DEGs specific to WT#67^flg/m^ revealed an enrichment for GO categories associated with photosynthesis and chloroplast‐related processes (Figure 4D), with DEGs in these GO categories being downregulated in wild‐type seedlings in response to flg22 (Figure S2C). A GO analysis for cellular components did not retrieve any statistically significant enrichments. The 1059 genes differentially expressed in *Br prt6.2/3#68* ^flg/m^ only were, in contrast, not found to be enriched for genes associated with specific GO Biological Processes. However, a GO Cellular Component enrichment analysis revealed an overrepresentation of genes associated with specific compartments such as the “Golgi apparatus” and with the movement of proteins and molecules, including genes associated with “intracellular vesicle” for example. This points to (i) a potential role of Br PRT6 enzymes in the regulation of chloroplast‐related processes in wild‐type *B. rapa* treated with flg22 and (ii) a potential dysfunction of protein transport in *Br prt6.2/3* seedlings in response to flg22 compared to the wild type.

We next tested the response of the *Br prt6.2/3* mutant to flg22 in a growth inhibition assay that involves 7‐day cocultivation of 3‐day‐old seedlings with 100‐nM flg22 and found that there were not statistically significant differences between the wild‐type lines and the mutants (Figure 4E). Similarly, apoplastic ROS production in response to flg22 treatment was not different between the wild type and the *Br prt6.2/3* mutant lines (Figure 4F). Finally, we assessed whether the *Br prt6.2/3* mutants exhibited a different level of resistance to the necrotrophic fungus *Sclerotinia sclerotiorum* but found no differences in lesion size area between the genotypes tested (Figure 4G). In sum, the flg22 response transcriptional program of *Br prt6.2/3* exhibits differences compared to that of wild‐type *B. rapa* seedlings, but this does not translate into defects in other PTI assays or in response to *S. sclerotiorum*.

## 4 Discussion

The Arg/N‐degron pathway has been studied in detail in the model plant *A. thaliana* , but much less is known about the functions of its enzymatic components in crop species, especially those of the Brassicaceae family such as the diploid *B. rapa* , a close relative of the allotetraploid *B. napus* (oilseed rape). Here, we isolated and characterized the first *B. rapa* Arg/N‐degron pathway mutants from a TILLING collection (Stephenson et al. 2010), focusing on two enzymatic components of the Arg/N‐degron pathway, the Arg‐transferases ATE1/2, and the downstream E3 ubiquitin ligase PRT6 (Figure 1A). Strikingly, the *Br a1a2* double mutant exhibited early developmental arrest, which is in stark contrast to the mild developmental defects of Arabidopsis *ate1 ate2* double mutant plants (i.e., leaf morphology defects, early outgrowth of axillary meristems, phyllotaxis defects, and delayed leaf senescence) (Yoshida et al. 2002; Graciet et al. 2009). This phenotype also appears to be stronger than that of *ATE* knock out lines of *Physcomitrella patens* , which exhibited delayed development (Schuessele et al. 2016). Although it remains possible that another cosegregating mutation may contribute to the early developmental arrest phenotype observed, the isolation of single *Br ate1‐2* and *Br ate2‐2* mutant plants, as well as the use of segregating F3 populations from either *Br ate1‐2/+ ate2‐2* or *Br ate1‐2 ate2‐2/+* parents, strongly suggests an essential role of Arg‐transferases in the regulation of cellular processes at early stages of development, possibly in the shoot apical meristem. In Arabidopsis, the shoot meristem regulator ZPR2 is the only ZPR protein targeted for degradation by the Arg/N‐degron pathway in an oxygen dependent manner due to its N‐terminal Cys residue (Weits et al. 2019) (ZPR1/3/4 in Arabidopsis do not start with N‐terminal Cys). ZPR proteins interact with Class III homeodomain leucine zipper (HD‐ZIP III) transcription factors, including REVOLUTA (REV), PHABULOSA (PHB), and PHAVOLUTA (PHV) and negatively regulate their activity (Kim et al. 2008; Gruber et al. 2021). Hence, plants accumulating ZPR proteins, such as Arg/N‐degron pathway mutants, could show similar phenotypic defects as mutants with reduced HD‐ZIP III transcription factor activity. BLASTp analysis with Arabidopsis ZPR1/2/3/4 retrieved 11 putative ZPR protein homologs in *B. rapa* , four of which start with the Met‐Cys sequence, which is characteristic of oxygen‐dependent substrates of the Arg/N‐degron pathway (the initial Met residue is removed by methionine aminopeptidases, to expose Cys at the N‐terminus). This increased number of ZPR Arg/N‐degron substrates in *B. rapa* could result in a stronger dependency on the Arg/N‐degron pathway to regulate ZPR protein accumulation, which may trigger higher ZPR protein levels in *Br a1a2* than in an Arabidopsis *ate1 ate2* mutant. In turn, this could result in a stronger inhibition of HD‐ZIP III in *B. rapa* and a more severe phenotype similar to that of Arabidopsis *rev‐6 phb‐13* double mutant seedlings, which exhibit an early arrest of development (Prigge et al. 2005), a phenotype that is similar to that of *Br a1a2* double mutant plants. An additional, nonmutually exclusive, possibility is that the early developmental arrest of *Br a1a2* mutants is due to the accumulation of *B. rapa‐*specific Arg‐transferase substrates not present in Arabidopsis and whose removal is normally required for seedling development to continue. Interestingly, the developmental arrest phenotype of *Br a1a2* is reminiscent of the embryonic lethality phenotype of *ate1* mice, in which the only *Mm ATE1* gene is knocked out (Kwon et al. 2002; Brower and Varshavsky 2009), highlighting the essential roles of Arg‐transferases across a broader range of eukaryotes. Our observations with *Br a1a2* also illustrate the difficulties of translating results from a model plant such as Arabidopsis to crops (Roeder et al. 2025; Uauy et al. 2025).

To by‐pass the early developmental arrest phenotype of *Br a1a2*, which precluded further investigation of the Arg/N‐degron pathway, and to avoid working with segregating populations from *Br ate1‐2 ate2‐2/+* parents, we isolated a *Br prt6.2/3* double mutant line containing early stop codon mutations in the two (out of three) most strongly expressed *B. rapa* homologs of Arabidopsis *PRT6*. *Br prt6.2/3* mutants developed similarly to wild‐type plants, while showing increased stability of Asp‐LUC and Arg‐LUC reporters, indicating that overall PRT6 activity was sufficiently impaired to allow accumulation of Arg/N‐degron pathway substrates. This is in agreement with the constitutive upregulation of hypoxia‐response genes in *Br prt6.2/3*, likely due to the stabilization of the ERFVII transcription factors. Hence, the *Br prt6.2/3* lines could be a suitable tool to study the functions of the Arg/N‐degron pathway in *B. rapa* .

Our characterization of *Br prt6.2/3* lines focused on responses to (a)biotic stresses for which differences between Arabidopsis wild‐type and Arg/N‐degron pathway mutant have been published. Arabidopsis *prt6* mutants are more tolerant to hypoxia stress (Gibbs et al. 2011), as well as to submergence (Riber et al. 2015), although for the latter, contrasting observations were made (Licausi et al. 2011). Here, under the experimental conditions applied, *Br prt6.2/3* plants were more susceptible to waterlogging than the wild type (Figure 3A). Submergence and waterlogging assays yield variable results in general, with many different parameters contributing to differential outcomes (e.g., light/dark and humidity). Here, the observed sensitivity of *Br prt6.2/3* compared to the wild type may reflect an increased susceptibility of *Br prt6.2/3* roots to waterlogging stress, which may affect the ability of the mutants to maintain metabolic pathways and physiological processes. The sensitivity could also result from differences in the size of the root systems, although the latter could not be analyzed due to difficulties in maintaining an intact root system after waterlogging. Root growth survival assays are also more difficult to carry out with *B. rapa* due to the larger size of the seedlings/plants and the need to grow them in a vertical manner to allow the roots to grow along the medium. Another possibility is that carbon metabolism and starvation response in the context of waterlogging or hypoxia in *B. rapa* may be different from that of Arabidopsis. However, this is unlikely to be the case, as such differences have not been observed when comparing *B. napus* and Arabidopsis transcriptional responses to hypoxia, as well as sugar levels (Ambros et al. 2022). We sought to test the response of the *Br prt6.2/3* mutant to hypoxia. These tests also suggested that the *Br prt6.2/3* mutant is more susceptible to hypoxia, even though conflicting evidence was found between the two *Br prt6.2/3* lines used when measuring chlorophyll levels. Survival in such assays is often associated with sugar metabolism and the ability to withstand starvation; however, Arabidopsis and *B. napus* do not seem to show significant differences in this respect (Ambros et al. 2022), and a similar situation is likely the case with *B. rapa* . In Arabidopsis, *prt6* mutants have been shown to be more salt stress tolerant (Vicente et al. 2017), but *Br prt6.2/3* mutants behaved similarly to the wild type in our assays. One possibility is that the remaining functional allele, Br PRT6.1, is sufficient to mask a phenotype.

In Arabidopsis, Arg/N‐degron pathway mutants differ from wild type in terms of their defense response to a range of pathogens (de Marchi et al. 2016; Gravot et al. 2016; Vicente et al. 2019). We identified transcriptional differences in the response of the *Br prt6.2/3* mutant to the model PAMP flg22, specifically in terms of the regulation of photosynthesis‐related genes (which were enriched among wild type only DEGs) and of processes involved in cellular transport (enriched among DEGs specific to *Br prt6.2/3*). This suggests a potential role of the Arg/N‐degron pathway in the regulation of PTI, despite the lack of other PTI‐associated defects in the double mutant. We also tested the response of the *Br prt6.2/3* mutant to the necrotrophic fungal pathogen *S. sclerotinia* but did not observe differences compared to the wild type. This contrasts with the increased susceptibility of the Arabidopsis *ate1 ate2* mutant (de Marchi et al. 2016) and could be the result of the remaining activity of Br PRT6.1 in the double mutant.

In summary, the results from the Arabidopsis/ *B. rapa* comparative analyses reinforce the need to validate knowledge gained in model systems via direct experimentation in crop species (Roeder et al. 2025; Uauy et al. 2025). For example, the species‐specific functions of the Arg/N‐degron pathway identified here suggest a partial divergence of the physiological roles of the Arg/N‐degron pathway since the split of Arabidopsis from Brassicas 43 million years ago (Beilstein et al. 2010). As the Arg/N‐degron pathway components and structure appear to be well conserved, this likely reflects variability in each species in the substrate repertoire and/or in the regulation of pathways or targets downstream of Arg/N‐degron pathway substrates. Such differences could be driven by direct selective pressures at N‐termini (e.g., gain or loss of a destabilizing N‐terminal residue) or by species‐specific proteases that may generate destabilizing neo‐N‐termini after cleavage.

## Author Contributions

B.C.M., P.G., S.S., and E.G. designed the work, conducted experiments, analyzed data, and wrote the manuscript.

## Funding

This work was supported by the Science Foundation Ireland (SFI) (13/IA/1870), the Research Ireland (Researchirel) (20/FFP‐P/8433), and the Irish Research Council (IrishResearch) (GOIPG/2017/2).

## Conflicts of Interest

The authors declare no conflicts of interest.

## Peer Review

The peer review history for this article is available in the Supporting Information for this article.

## Supporting information


**Data S1:** Peer Review.

**Data S2:** Supporting Information.

**Data S3:** Supporting Information.

**Table S1:** List of *B. rapa* lines used and isolated in this study.
**Table S2:** List of oligonucleotides used in this study.
**Figure S1:** Single mutant *Br ate1‐2* and *Br ate2‐2* seedlings are similar to Ro18 wild‐type seedlings. Seeds of each genotype were sown on 0.5× MS agar supplemented with 0.5% sucrose. Seedlings were grown in continuous light for 6 days.
**Figure S2:** Supplemental analysis of RNA‐seq experiment with flg22. (A) RT‐qPCRs to verify induction of flg22 response genes after 1 h of treatment of 3‐day‐old *B. rapa* seedlings of different genotypes. Mean of 3 biological replicates with SEM. Statistical analysis: 2‐way ANOVA with Tukey's test. (B) Comparison of directionality and amplitude of gene expression changes amongst DEGs common to wild type and *Br prt6.2/3* mutants in response to flg22. (C) Gene expression changes as log_2_(fold change) of DEGs in wild‐type seedlings only that belong to the GO categories shown in Figure 4D.

## Data Availability

Raw and processed data are submitted to NCBI Gene Expression Omnibus under Accession Number GSE311966.
