# Supplementary material for: Functional Divergence of the Arg/N‐Degron Pathway Between the Crop Brassica rapa and the Model Plant Arabidopsis thaliana
Source: Plant Direct. 2026 Mar 19;10(3):e70158. doi: 10.1002/pld3.70158 (PMC13103730; doi:10.1002/pld3.70158)
Supplement: Supplementary file 1 — Data S1: Peer Review. [file PLD3-10-e70158-s001.pdf]

Functional divergence of the Arg/N-degron pathway between the crop Brassica rapa and the model plant Arabidopsis thaliana

Decision Letter Round 1:

January 6, 2026

Prof. Emmanuelle Graciet

Maynooth University Department of Biology

Maynooth, N/A

Ireland

RE: Functional divergence of the Arg/N-degron pathway between the crop Brassica rapa and the model plant Arabidopsis thaliana

Dear Dr. Graciet:

Thank you for submitting to Plant Direct. All required reviews have been returned and we have now finished our evaluation of your manuscript. In light of the reviewers' comments, a few further revisions are needed before the paper can be accepted for publication in Plant Direct. In particular, it will be important to provide a more nuanced assessment of the potential essential role of ATEs and the caveats of background mutations in the TILLING lines.

Please view the reviewers' comments below and use their suggestions as a guide while you work on your revision.

When uploading the revised version of this article, please be sure to include the following:

-A word document that contains your response to the reviewers. You should respond to each reviewer comment and note the changes made to the manuscript. If you do not agree with a reviewer's comment and choose not to make a suggested revision, please explain why. Please try to provide as complete an answer as possible to each reviewer's criticisms.

-A tracked changes document with each change highlighted

- A clean version of the latest version of the manuscript

To upload your revision, please click the link below.

https://plantdirect.msubmit.net/cgi-bin/main.plex?el=A6Lr3qI6A5lpO6I7A9ftddDlrfD95sJm1Px5a4PQZ

In order to provide as timely a service as possible, we ask that your revision is resubmitted within three months after receipt of this request. If an extension is needed, please send a request, along with a brief explanation, to the editorial office at plantdirect@wiley.com .

Please note that, in addition to publishing reviewer comments, the author's responses to review comments will also be published alongside the final version of the paper. If you would not like the author's responses to be published, please contact the editorial office at plantdirect@wiley.com .

Thank you very much for giving us an opportunity to review your work. I look forward to receiving the next version.

Sincerely,

Eva Farre

Gustavo MacIntosh

Editor, Plant Direct

-- Reviewer comments:

Reviewer #1:

The manuscript on functional divergence of the Arg/N-degron pathway between the crop Brassica rapa and the model plant Arabidopsis thaliana reports on mutants in N-degron components of B. rapa.

The manuscript is well written and consistent, and reports an interesting characterization of the mutants in PRT6 homologs, including transcript analysis based on RNA-seq.

I have one major question: Did the authors obtain mutants B. rapa homozygous for the Br ate2-2 mutation ? The reason for this question is that with the data presented, it is possible that a mutation in an essential gene linked to Br ATE2 causes the lethal phenotype. In that case, a single mutant "ate2-2" would also be lethal. However, if

indeed the KO of the two ATE genes is non-viable, one might expect that the single ate2-2 mutant is viable and due to the presence of the functional ATE1 gene, and it may have a mild phenotype. If the authors do not have such data, they should mention this intellectual loophole in their chain of arguments that ATE1/2 knockout is lethal, unlike the situation in Arabidopsis. (Another, high-quality, support for their argument would be if an ectopic ATE2 or ATE1 gene brought in by transformation would complement the double mutant lethality, but this would really be a lot of work, by far exceeding usual revision efforts)

Minor:

Lines 646,653, 655: incomplete references

Line 416/417: treating … seedlings …with … (treatment?) …

Line 451: … fell into a wide (range?) of protein functions …

Line 532: … roots to grow(n?) along the medium.

Reviewer #2:

The N-degron pathway has been characterised extensively in the model plant Arabidopsis and shown to play important roles in responses to abiotic and biotic stresses as well as plant development. This manuscript explores the N-degron pathway in a related crop, Brassica rapa. Whilst some aspects are conserved between Arabidopsis and B.rapa, notable differences were revealed, underscoring the importance of working directly in crop species. The manuscript is concise and clearly written.

The authors isolated TILLING mutants affected in two components of the Arg/N-degron pathway: arginyl transferase enzymes (ATEs) and the N-recognin, PRT6. As B.rapa has two ATE genes and three PRT6 homologues, TILLING lines were crossed to create double mutants. Interestingly, the double ate mutant line was arrested in seedling development, whereas it can complete a full life cycle in Arabidopsis. For this reason, the authors elected not to create a triple PRT6 knockout line but rather focused on the most highly expressed PRT6 gene (Br PRT6.2) and made a double mutant with a line affected in BrPRT6.3. I am curious whether the authors attempted to generate a line with mutations in all three genes and whether this had a similar phenotype to the Br ATE1/2 line? This is of interest because the Arabidopsis ate1/2 mutant has a more severe phenotype than the prt6 mutant (as shown by the senior author in 2009). Please note that I do not expect that the authors should create a triple mutant if they have not done so. However, it is important to note that the residual PRT6 activity from Br PRT1

may mask phenotypes, as the authors discuss in the context tht B. rapa double mutants did not show differential response to salt stress or to biotic stress compared to the wild type.

TILLING lines typically have numerous background mutations which should be removed by backcrossing as far as linkage allows. However, this is not always practical in the lifetime of a project, especially given the long generation time of crops. Can the authors please at least comment on the mutation rate in the B.rapa TILLING population and the theoretical number of backcrosses needed to clean up the lines? I note that the authors did obtain a "wild type" control from the segregating F2 population control for the presence and potential effects of segregating background mutations that may have been retained despite back-crossing with Ro18.

Please could the authors state the background gene/transcript set used for GO terms enrichment and confirm that they have followed best practice as outlined in https://doi.org/10.1371/journal.pcbi.1009935 ?

Unlike Arabidopsis prt6 mutants, the B. rapa prt6 double mutants were more sensitive to waterlogging. Potential reasons for this are discussed in the manuscript but one possibility is that sensitivity to waterlogging could be related to a smaller root system. Although it is harder to measure root growth in B. rapa, roots could be weighed to explore this possibility.

A minor point, please use the abbreviation ERFVII, not ERF-VII.

Decision Letter Round 2:

February 13, 2026

Prof. Emmanuelle Graciet

Maynooth University Department of Biology

Maynooth, N/A

Ireland

RE: Functional divergence of the Arg/N-degron pathway between the crop Brassica rapa and the model plant Arabidopsis thaliana

Dear Dr. Graciet:

I am pleased to inform you that your manuscript "Functional divergence of the Arg/N-degron pathway between the crop Brassica rapa and the model plant Arabidopsis thaliana" has been accepted for publication in Plant Direct. However, we please request that you clarify a last question from Reviewer #1 and make any necessary changes.

When uploading the revised version of this article, please be sure to include the following:

-A word document that contains your response to the reviewers. You should respond to each reviewer comment and note the changes made to the manuscript. If you do not agree with a reviewer's comment and choose not to make a suggested revision, please explain why. Please try to provide as complete an answer as possible to each reviewer's criticisms.

-A tracked changes document with each change highlighted

- A clean version of the latest version of the manuscript

To upload your revision, please click the link below.

https://plantdirect.msubmit.net/cgi-bin/main.plex?el=A6Lr7qI3B6lpO1I1A9ftddDlrfD95sJm1Px5a4PQZ

In order to provide as timely a service as possible, we ask that your revision is resubmitted within three months after receipt of this request. If an extension is needed, please send a request, along with a brief explanation, to the editorial office at plantdirect@wiley.com .

Please note that, in addition to publishing reviewer comments, the author's responses to review comments will also be published alongside the final version of the paper. If you would not like the author's responses to be published, please contact the editorial office at plantdirect@wiley.com .

Thank you very much for giving us an opportunity to review your work. I look forward to receiving the final version of the manuscript.

Sincerely,

Eva Farre

Gustavo MacIntosh

Editor, Plant Direct

-- Editor comments:

-- Reviewer comments:

Reviewer #1:

The authors have considered reviewer requests.

Minor:

p13, line 4: Br ate2-2 single mutants

instead of ate1-2 single mutants ?

Reviewer #2:

I am satisfied that the authors have addressed my comments.

Decision letter Round 3:

February 27, 2026

Prof. Emmanuelle Graciet

Maynooth University Department of Biology

Maynooth, N/A

Ireland

MSID: 2025-01864R2

MS TITLE: Functional divergence of the Arg/N-degron pathway between the crop Brassica rapa and the model plant Arabidopsis thaliana

Dear Dr. Graciet:

I am pleased to inform you that your manuscript "Functional divergence of the Arg/N-degron pathway between the crop Brassica rapa and the model plant Arabidopsis thaliana" has been accepted for publication in Plant Direct.

Your article will appear online in the next available issue of Plant Direct. To ensure your article gets published as quickly as possible, please pay attention to the steps detailed below. We have found that most of the delays happen at this stage, especially at the payment stage, so please respond as quickly as possible when prompted.

License Agreement: Once your article has been accepted it will move to Production and undergo admin and file checking - you may receive an email with any queries we have at this stage. When all required items are received by the publisher and queries resolved, the corresponding author will receive an email from Wiley's Author Services system which will ask them to log on at https://authorservices.wiley.com/bauthor and will present them with the appropriate license for completion. Your article cannot be published until both the signed license agreement and payment of the article fee have been received.

Payment of the Open Access Article Publication Fee: All articles published in Plant Direct are fully open access: immediately and freely available to read, download and share. Plant Direct charges a publication fee to cover the publication costs. The corresponding author for this manuscript should have already received a quote from the payments team (cs-openaccess@wiley.com) with the estimated article publication fee; please email cs-openaccess@wiley.com if this has not been received. The corresponding author should log on to the Wiley Author Services site, where the publication fee can be paid by credit card or an invoice. Pro Forma can also be requested. Payment of the publication charge must be received before the article will be published online.

Proofs: You will have the opportunity to look over your paper once more when you receive the author proofs for your article. The proofs will be with you in approximately two weeks. Please note that, in addition to publishing reviewer comments, the author's responses to review comments will also be published alongside the final version of the paper. If you would not like the author's responses to be published, please contact the editorial office at plantdirect@wiley.com .

Promotion of your article: You can help your research get the attention it deserves! Check out Wiley's free Promotion Guide for best-practice recommendations for promoting your work at www.wileyauthors.com/eeo/guide. And learn more about Wiley Editing Services which offers professional video, design, and writing services to create shareable video abstracts, infographics, conference posters, lay summaries, and research news stories for your research at www.wileyauthors.com/eeo/promotion.

Thank you again for your contribution to Plant Direct. If you have any questions, feel free to contact the editorial office at plantdirect@wiley.com .

Sincerely,

Eva Farre

Editor, Plant Direct
